# Discrepancy Between Conventional Coagulation Tests and Thromboelastography During the Early Postoperative Phase of Liver Resection in Neoplastic Patients: A Prospective Study Using the New-Generation TEG^®^6s

**DOI:** 10.3390/jcm14092866

**Published:** 2025-04-22

**Authors:** Rita Gaspari, Paola Aceto, Simone Carelli, Alfonso Wolfango Avolio, Maria Grazia Bocci, Stefania Postorino, Giorgia Spinazzola, Mariagiovanna Caporale, Felice Giuliante, Massimo Antonelli

**Affiliations:** 1 Department of Basic Biotechnological Sciences, Intensive Care Peri-Operative Clinics, Università Cattolica del Sacro Cuore, 00168 Rome, Italy; rita.gaspari@unicatt.it (R.G.); massimo.antonelli@unicatt.it (M.A.); 2 Department of Emergency, Anesthesiological and Reanimation Sciences, Fondazione Policlinico Universitario Agostino Gemelli IRCCS, 00168 Rome, Italy; simone.carelli@policlinicogemelli.it (S.C.); stefania.postorino@policlinicogemelli.it (S.P.); giorgia.spinazzola@policlinicogemelli.it (G.S.);; 3 Department of Translational Medicine and Surgery, Università Cattolica del Sacro Cuore, 00168 Rome, Italyfelice.giuliante@unicatt.it (F.G.); 4 General Surgery and Transplantation Unit, Department of Medical and Surgical Sciences, Fondazione Policlinico Universitario Agostino Gemelli IRCCS, 00168 Rome, Italy; 5 UOC Resuscitation, Istituto Nazionale per le Malattie Infettive L. Spallanzani IRCCS, 00149 Rome, Italy; mariagrazia.bocci@inmi.it; 6 Hepatobiliary Surgery Unit, Department of Medical and Surgical Sciences, Fondazione Policlinico Universitario Agostino Gemelli IRCCS, 00168 Rome, Italy

**Keywords:** coagulation profile, liver resection, coagulation tests, viscoelastic tests

## Abstract

**Background**: Thromboelastography-6s (TEG^®^6s), a novel device developed to assess coagulation status, presents advantages such as less frequent calibration, ease of use, and greater stability against movements compared to the previous system (TEG5000). This is the first study in the literature to compare coagulation profiles in the early postoperative period of liver resection (LR) using conventional coagulation tests (CCTs) and TEG^®^6s. **Methods**: Forty-six adult patients admitted to the ICU post-surgery after elective LR for malignancy were included. CCTs were used to classify patients into hypocoagulable (HCG) (platelet count < 80 × 10^9^/L, international normalized ratio ≥ 1.4, or activated partial thromboplastin time > 38 s) and normocoagulable (all other cases) groups. Mann–Whitney tests, Spearman’s correlation, and linear regression were used. **Results**: On ICU admission, nineteen (41.3%) patients had a hypocoagulable profile based on CCTs, but only two (10.5%) of them were rated as hypocoagulable by TEG (*p* = 0.165). Intraoperatively, HCG patients experienced higher estimated blood loss (EBL) (*p* = 0.002); they required more fluids (*p* = 0.019), and more of them received red blood cell transfusions (*p* = 0.025). They also had higher postoperative arterial lactate levels (*p* = 0.036). Postoperative 12 h EBL was similar in the two groups (around 150 mL). The ICU stay was longer for HCG group (*p* = 0.010). Weak associations were observed between TEG/CCTs measures of coagulation initiation [e.g., between R time citrated rapid TEG, and international normalized ratio (r^2^ = 0.448; *p* < 0.001)], clot formation [i.e., between conventional fibrinogen value using Clauss method and α-angle citrated rapid TEG (r^2^ = 0.542; *p* < 0.001)], and clot strength [e.g., between conventional fibrinogen and citrated kaolin maximum amplitude (r^2^ = 0.484; *p* < 0.001)]. **Conclusions**: CCTs revealed hypocoagulability that was not confirmed by TEG^®^6s. However, the thromboelastography coagulation profile was more consistent with the detected non-relevant postoperative bleeding.

## 1. Introduction

Liver resection (LR) is commonly used for patients with primary or metastatic liver cancer and benign liver lesions [1,2,3]. In postoperative care, LR may enhance or induce coagulation derangement that can be challenging to predict and manage. Factors that may contribute to this coagulation imbalance include pre-existing liver disease, reduced residual liver volume, intraoperative blood loss, and intraoperative ischemia–reperfusion injury. All these factors can impair the hepatic synthesis of procoagulant factors and lead to transient hypocoagulability. Conversely, liver cancer is a factor that promotes hypercoagulability or counteracts the tendency toward hypocoagulability. Hypercoagulability may occur due to a concomitant decrease in the concentration of anticoagulant factors produced by the liver and/or the reduced clearance of activated coagulation factors (VIII, von Willebrand factor and tissue factors) [4,5,6,7]. Previous studies have shown that patients undergoing LR may present significant changes in conventional coagulation tests (CCTs), such as prothrombin time/international normalized ratio (PT/INR) or activated partial thromboplastin time (aPTT), suggesting hypocoagulability [8,9,10]. This condition has led many physicians to transfuse large volumes of fresh frozen plasma in the postoperative period, exposing patients to transfusion-associated circulatory overload and transfusion-associated acute lung injury [11]. In addition, LR patients may develop pulmonary embolism and deep venous thrombosis due to the overestimation of the bleeding risk and subsequent delay in the onset of thromboprophylaxis [12]. So far, point-of-care viscoelastic tests (VETs) such as thromboelastography (TEG) and rotational thromboelastometry have been developed to assess coagulopathy in the postoperative period [9,10,13,14,15,16,17]. Compared to CCTs, VETs have an easier process and faster response time since they are performed on all the blood rather than on plasma alone. The results are obtained in real time, with specific trace parameters reflecting normal or altered coagulation processes [9,10,13,14,15,16,17]. More recently, a novel, fully automated thromboelastography device, TEG^®^6s (Haemonetics, Braintree, MA, USA) [18], was introduced, which uses resonant frequency analysis and a multi-channel cartridge housing to achieve greater speed, accuracy, and reproducibility by simply inserting a cartridge and adding blood. It was introduced to assess coagulation status during specific procedures at risk of bleeding, such as cardiac, obstetric, and trauma surgery and liver transplantation [19,20,21,22]. This device offers advantages over TEG 5000, including less frequent calibration requirements, ease of use, and reduced sensitivity to movement. The performance of TEG^®^6s in the early postoperative period of cancer patients undergoing LR has not been studied. This prospective observational study aimed to investigate the coagulation profile in the early postoperative period of patients undergoing oncological LR, using CCTs and TEG^®^6s. We hypothesized that the hypocoagulability assessed by CCTs could have a minimal impact on the TEG tracing. We also compared TEG^®^6s with CCT parameters to assess the degree of correlation between the two approaches. Specifically, we investigated the correlations between CCTs and TEG parameters involved in coagulation initiation, clot formation, clot strength, and fibrinolysis.

## 2. Materials and Methods

### 2.1. Study Design and Population

This prospective observational cohort study was performed at the Fondazione Policlinico Universitario A. Gemelli IRCCS (Rome, Italy) between April 2023 and October 2023. Inclusion criteria were age ≥ 18 years, elective liver surgery for primary or secondary malignant liver disease, and admission to the intensive care unit (ICU) at the end of surgery. Exclusion criteria included urgent liver surgery and reoperation due to postoperative complications that required re-admission to the ICU (e.g., bleeding). This study was approved by the local ethics committee (ID number: 5492) on 16 March 2023 and conducted following the Declaration of Helsinki of the World Medical Association revised in 2013 for experiments involving humans. Written informed consent was obtained from all patients.

### 2.2. Conventional Coagulation Tests

CCTs included INR, PT, aPTT assays, fibrinogen levels measured by Clauss methods or conventional fibrinogen, D-dimer levels, antithrombin III (AT III) percent activity, and platelet count.

According to Ramspot et al. [23], the patients were divided into two groups based on the CCTs: the hypocoagulable (HCG) group and the normocoagulable (NCG) group. The hypocoagulable status was defined by platelet count < 80 × 10^9^/L, INR ≥ 1.4, or aPTT > 38 s. In the absence of these alterations, all remaining cases were defined as normocoagulable (NCG).

### 2.3. Thromboelastography 6s Analysis

TEG^®^6s parameters, representing different aspects of hemostasis, resulted from four-channel cartridge analysis. Citrated kaolin TEG (CK), citrated kaolin TEG with heparinase (CKH), which neutralizes the effects of heparin, and citrated rapid TEG (CRT), an accelerated assay with kaolin and the tissue factor, were performed to analyze global hemostasis results whilst citrated TEG functional fibrinogen (CFF)—which uses the tissue factor as a clotting activator and GpIIb/IIIa inhibitors to neutralize platelet function—was performed to assess the contribution of fibrinogen to clot strength [7,8,9,10,11,12,13,14,15,16,17,18,19,20,21].

TEG^®^6s tracings and derivative parameters include reaction time (R), the time from the start of the assay to the point where clot strength reaches an amplitude of 2 mm; clotting time (K), the time from the end of the reaction time until the amplitude reaches 20 mm; the maximum amplitude (MA), representing the absolute strength or stability of the clot; and the alpha (α) angle, the angle formed by the slope of a tangent line from the tracing at the midpoint between the R and the K time, which indicates the strength of the clot. The percentage reduction in the area under the TEG curve from the MA to 30 min after the MA indicates the status of fibrinolysis (30 min clot lysis—LY30).

Based on single TEG^®^6s parameters, hypocoagulability was defined as prolonged R and K times and/or a decreased α-angle and MA below the reference values. Conversely, hypercoagulability was defined as shortened R and K times and/or increased α-angle and MA values above the reference values [24].

### 2.4. Anesthesia and Perioperative Management

Based on the current guidelines, preoperative antiplatelet or anticoagulant therapy was discontinued before surgery [25].

The anesthetic protocol was standardized. Anesthesia was induced with propofol (2 mg/kg) and fentanyl (3 mcg/kg) and maintained with sevoflurane (age-adjusted 1 minimum alveolar concentration in a 40% air–oxygen mixture) and the infusion of remifentanil (dose ranging from 0.05 to 0.2 mcg/kg/min). Muscle relaxation was induced with rocuronium (0.6 mg/kg) and maintained with additional boluses (0.15 mg/kg). Mechanical ventilation was set to keep end-tidal carbon dioxide between 35 and 45 mmHg (tidal volume of 6–8 mL with ideal body weight) and a positive end-expiratory pressure of 5 mmHg was applied. Hemodynamic monitoring included observations of heart rate and electrocardiograms (D2-V5 derivations), pulse oximetry, and invasive pressure through radial artery cannulation. At the time of skin incision, all patients received an infusion of Rehydrating Electrolyte Solution (RES) at 3 mL/kg/h (i.e., basal fluid infusion). Hemodynamics were managed using the FloTracTM/VigileoTM system (Vigilance Monitor Edwards Life Sciences, Irvine, CA, USA), which uses a pulse contour analysis to calculate the cardiac index (CI) [26]. A fluid bolus (250 mL) of RES was administered when the mean arterial pressure (MAP) was <65 mmHg, CI was <2.5 L/min/m^2^, and stroke volume variation (SVV) was >13%. In the case of an MAP < 65 mmHg and CI ≥ 2.5 L/min/m^2^, regardless of SVV, continuous norepinephrine infusion was started at a dose of 0.1 mcg/kg/min and titrated to achieve an MAP ≥ 65 mmHg; after reaching an MAP ≥ 65 mmHg, SVV was optimized (if ≥13) with RES bolus after the end of the liver parenchymal resection phase or during the liver resection phase only in the case of urine output < 1 mL/kg/h. Continuous dobutamine infusion was considered the first-line intervention when the CI < 2.5 L/min/m^2^, MAP < 65 mmHg, and SVV ≤ 13%. Human albumin (20 g/L) was administered only to treat clinically documented hypoalbuminemia (<2.8 g/dL). Packed red blood cells (PRBCs) were transfused to maintain a hemoglobin concentration of ≥8 g/dL (≥9 g/dL in patients with a history of ischemic heart disease). A forced-air warming system (Bair Hugger Model 505, Arizant Healthcare Inc., St. Paul, MN, USA) and a fluid warming device (enFlowR, BD, Franklin Lakes, NJ, USA) were used to maintain patients’ temperature within the normal range. At the end of surgery, all patients, still sedated, intubated, and mechanically ventilated, were transferred to the ICU, where they were gradually awakened. Sequential pneumatic compression was applied to the lower limbs during and after surgery until the patient was mobilized to prevent the risk of thrombosis. According to Anderson et al. [27], thromboprophylaxis also included a daily subcutaneous injection of low-molecular-weight heparin 4000 internation units starting 12 h after the end of surgery in the absence of bleeding.

### 2.5. Types of Resections

LR was defined according to the International Hepato-Pancreato-Biliary Association terminology [28]. Resections ≥ 3 segments were classified as major hepatectomies. Minor complex hepatectomies included ≥3 parenchymal-sparing liver resections for metastases. During surgery (either laparoscopic or laparotomic), transient occlusions of the inflow (hepatic artery and portal vein), i.e., the Pringle maneuver, were generally performed to reduce bleeding in the resection plane.

### 2.6. Postoperative Care in ICU

Blood samples for standard laboratory parameters were collected in Vacutainer tubes from the arterial line on ICU arrival. Two blood citrate samples (3 mL, sodium citrate solution, Vacuette^®^ blood tubes, Greiner Bio-One, Kremsmünster, Austria) were obtained simultaneously for CCTs and TEG^®^6s assessment at ICU admission. The sample for TEG was analyzed within 30 min after blood collection, while the sample for CCTs was sent to the laboratory. According to internal guidelines, patients were discharged from the ICU to the ward when they no longer required multiparameter monitoring and intensive care [29,30].

### 2.7. Data Collection

Demographic data included age, gender, the body mass index, the American Society of Anaesthesiology (ASA) physical status, comorbidities, preoperative anticoagulant/antiaggregant therapy, and preoperative hemoglobin. The histology of resected liver tissue (presence of cirrhosis) and etiology of the liver nodule were recorded for each patient.

Intraoperative parameters included the surgical approach (open/laparoscopic), the extent of hepatectomy, surgical time, the cumulative duration of the Pringle maneuver, estimated blood loss (EBL), the number of patients requiring PRBCs, fresh frozen plasma (FFP) and platelets, administered fluids, and need for vasopressors. Coagulation data and arterial lactate concentration were recorded at ICU admission. The arterial lactate concentration was measured in blood samples collected from the radial artery using heparinized syringes at 37 °C (Nova Biomedical Corporation, Waltham, MA, USA). The number of patients requiring PRBCs, FFP, platelets, administered fluids, EBL, fluid infusion, arterial lactate, and vasopressor use was also recorded during the first 12 h after ICU admission.

The ICU length of stay, ICU mortality, and thromboembolic events were also registered after 30 days.

### 2.8. Statistical Analysis

Continuous variables were expressed as the median and interquartile range [25th–75th percentile] and compared using the Mann–Whitney test. Categorical variables were given as proportions and analyzed with the chi-square test or Fisher’s exact test, as appropriate.

A descriptive analysis was performed to classify patients according to the presence of a hypocoagulable or normocoagulable pattern based on CCTs.

Continuous data were analyzed by computing Spearman’s correlation coefficients with their significance level. The correlation coefficient ranges were defined as follows: r < 0.3, weak; 0.7 < r ≥ 0.3, moderate; and r ≥ 0.7, strong correlation. Linear regression analysis was performed in cases of significant correlations (*p* < 0.05).

Two-tailed significance tests and a significance level of 0.05 were used. All statistical analyses were performed by using IBM SPSS version 28.0 (IBM Corp., Armonk, NY, USA).

## 3. Results

### 3.1. Patients’ Characteristics

**Figure 1:**
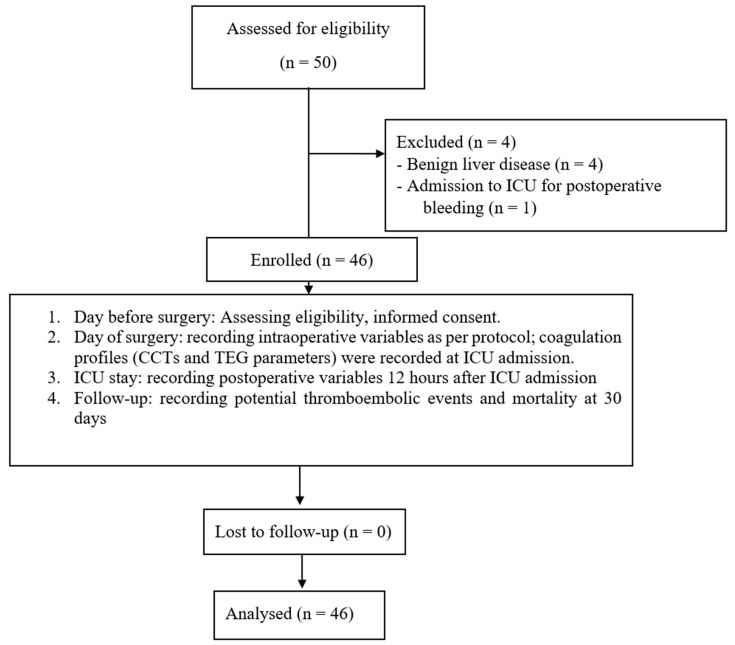
Study flow diagram with description of study design. CCTs, conventional coagulation tests; TEG, thromboelastography; ICU, intensive care unit.

Fifty patients underwent LR, and 46 patients were enrolled in this study (Figure 1). The study population consisted of 30 males (65.2%) and 16 females (34.8%); the median age was 70 years [59–75].

The most common indication was metastatic colorectal cancer (*n* = 26; 56.5%). Hepatocellular carcinoma was the second most common indication (*n* = 6; 13.0%), followed by cholangiocarcinoma (*n* = 4; 8.7%). Overall, seven patients (15.2%) also had evidence of cirrhosis (based on histological examination), and all of them were in the Child–Turcotte–Pugh class A. The distribution of cirrhosis did not vary between the two groups (*p* = 0.682).

The most common ASA class was class 2 (34 of 46 patients, 73.9%). Twenty-seven of the forty-six patients (58.7%) underwent minor LR, thirteen patients (28.3%) underwent major LR, and the remaining six patients (13.0%) underwent minor complex LR. Open surgery was performed in 25 patients (54.3%), whilst laparoscopy was used in the remaining 21 patients (45.6%). None of the patients received anti-fibrinolytic drugs.

### 3.2. Coagulation Data

On admission to the ICU, 19 patients (41.3%) had a hypocoagulable profile, and 27 patients (58.7%) showed a normal coagulable profile (Table 1).

Only two patients (both with decreased α-angle and MA) in the HCG group (10.5%) and no patient in the NGG group presented a hypocoagulable profile according to TEG parameters (*p* = 0.165). In the NCG group, only one case showed a hypercoagulable profile (increased α-angle and MA). There was a significant difference in intraoperative EBL between the two groups. Specifically, the HCG group had a higher EBL than the NCG group (900 mL [500–1400] vs. 400 mL [300–700]; *p* = 0.002).

In addition, the HCG group received more intraoperative fluids than the NCG group (4500 mL [5500–6000] vs. 3500 mL [3000–4010]; *p* = 0.019). A higher percentage of HCG patients received PRBC transfusions during surgery compared to NCG patients (10 [53%] vs. 5 [19%]; *p* = 0.025).

The HCG group had significantly lower AT III levels (49 [40–53] vs. 58 [51–72]; *p* = 0.002) and higher arterial lactate levels (6.3 [4.1–11.2] vs. 5.1 [2.5–6.6]; *p* = 0.036).

### 3.3. Clinical Data

During the first 12 h of the ICU stay, the median [IQR] EBL was 150 [100–200] mL in the entire population, and no significant differences were found between the two groups (*p* = 0.099). The length of the ICU stay was longer in the HCG than in the NCG group (*p* = 0.010). There were no hospital deaths in the first 30 days in either group. During the first 30 days, there was a higher incidence of venous thromboembolic events in the HCG compared to the NCG group, although the difference did not reach statistical significance (Table 2).

### 3.4. Correlations Between CCTs and TEG Parameters

Coagulation initiation: CRT-R showed moderate correlations with the INR (r = 0.647; *p* < 0.001) and with aPTT (r = 0.376; *p* = 0.011).

Clot formation: no correlations were found between CK-K and CRT-K and the INR and/or aPTT.

Clot strength: Moderate correlation levels were found between platelets and the CK-α-angle (r = 0.516; *p* < 0.001), CK-MA (r = 0.640; *p* < 0.001), CRT-α-angle (r = 0.413; *p* = 0.005), and CRT-MA (r = 0.618; *p* < 0.001). Moderate correlation levels were found between conventional fibrinogen levels and the CK-α-angle (r = 0.501; *p* < 0.001), CK-MA (r = 0.662; *p* < 0.001), CRT-α-angle (r = 0.759; *p* < 0.001), CRT-MA (r = 0.699; *p* < 0.001), CFF-MA (r = 0.683; *p* < 0.001), and CFF-A10 (r = 0.751; *p* < 0.001) (Table 3).

There were no associations between TEG and D-dimer levels.

### 3.5. Linear Regression

Weak associations were observed between TEG/CCT measures of coagulation initiation [e.g., between CRT-R and INR (r^2^ = 0.448; *p* < 0.001)], clot formation [i.e., between conventional fibrinogen level and CRT-α-angle (r^2^ = 0.542; *p* < 0.001)], and clot strength [i.e., between conventional fibrinogen levels and CK-MA (r^2^ = 0.484; *p* < 0.001)]; between conventional fibrinogen levels and CRT-MA (r^2^ = 0.526; *p* < 0.001); between conventional fibrinogen levels and CFF-MA (r^2^ = 0.515; *p* < 0.001); between conventional fibrinogen levels and CFF-A10 (r^2^ = 0.598; *p* < 0.001); between platelets and CK-MA (r^2^ = 0.419; *p* < 0.001); and between platelets and the CRT-α-angle (r^2^ = 0.371; *p* < 0.001) (see Table 3).

## 4. Discussion

In the past, the management of coagulation after liver resection has been a subject of controversy. Currently, several authors suggest that these patients are normo- or hypercoagulable, and much of the evidence is based on viscoelastic testing [9,16,31,32]. Nevertheless, there are cases with clinical suspicion of coagulopathy, especially in the presence of blood loss. To date, there is a lack of evidence regarding the best method to monitor coagulation status in this setting. To our knowledge, this is the first prospective study conducted to analyze coagulation profiles using the new thromboelastography (TEG^®^6s) and its performance in identifying hypocoagulation as assessed by conventional coagulation tests in patients undergoing elective liver resection for primary or metastatic cancer.

In this study, only 10.5% of patients exhibiting a hypocoagulable status according to CCTs were also identified as hypocoagulable by TEG. No differences were observed between patients with hypocoagulable and normocoagulable profiles regarding preoperative variables and some intraoperative parameters such as the duration of surgery, vascular clamping, the extent of parenchymal resection, and the type of surgical approach (open or laparoscopic). In contrast, we found that hypocoagulant patients experienced greater intraoperative blood loss, needed more volume resuscitation, and often required a higher rate of packed red blood cell transfusions compared to patients with normal conventional coagulation tests. Accordingly, they had significantly lower AT III levels and higher lactate values at ICU admission. As reported by other authors [5], coagulopathy can develop during major surgery due to excessive blood loss and subsequent consumption of coagulation factors and hemodilution. The correction of blood loss with packed red blood cells and fluids reduces plasma levels of most hemostatic elements, resulting in dilutional coagulopathy proportional to the degree of hemodilution reflected by conventional coagulation tests. We also observed a greater reduction in conventional fibrinogen levels and anticoagulant factors such as antithrombin III in the hypocoagulant group rather than in the normocoagulant group. The hypocoagulable state identified by CCTs does not indicate a true increased risk of bleeding. As reported by many authors, major abdominal surgery, including liver surgery, results in the decreased clearance of activated coagulation factors (factor VIII and von Willebrand factor) [4,6,7,8], which, together with blood loss, contributes to the development of a potentially prothrombotic state. The normality of TEG^®^6s in most of our patients who had a hypocoagulable state according to CCTs would suggest that the hemostatic balance is maintained by a simultaneous dilution of pro- and anticoagulant factors [10]. This ‘re-balancing of hemostasis’ may account for the lower incidence of postoperative bleeding in our population compared to the occurrence of thromboembolic complications. CCTs are routinely used in clinical practice to start thromboprophylaxis, whilst a delay in initiation may lead to thromboembolic events. Within this context, the question of when to start thromboprophylaxis based on laboratory data or TEG normality remains unresolved. We observed three thrombotic events, but our study population was not large enough to establish an association between coagulation profiles and thrombotic events.

The discrepancy between conventional tests and thromboelastographic variables found in our study is consistent with the results shown by other authors. De Pietri et al. [16] observed a hypocoagulable profile in the postoperative period of hepatic oncologic surgery, according to conventional tests, whereas the thromboelastographic tracings outlined a state of normocoagulability. Similarly, Tanner et al. [31], Louis et al. [9], and Gordon et al. [32] reported that the increase in prothrombin time/the INR is often not associated with a hypocoagulable state, while thromboelastography is more accurate in determining a true coagulation state in liver resection patients. Cerutti et al. [33], in the perioperative period of liver transplantation, described a hypercoagulable state on thromboelastography despite routine tests suggesting hypocoagulability. Notably, all these studies were performed using the old TEG technology, which requires the manual processing of samples and longer assay times.

In our population, associations were observed—although less consistent than those in acutely ill patients with chronic liver disease [34]—between TEG/CCTs measuring coagulation initiation (i.e., TEG R time and INR), clot formation (i.e., conventional fibrinogen level and TEG parameters), and clot strength (i.e., conventional fibrinogen levels and platelets with TEG parameters). Where detected, the poor correlations between CCTs and TEG^®^6s might suggest that the two methods measure different aspects of the coagulation process, making them complementary but not interchangeable. In contrast, inconsistent associations were found between fibrinolysis measures (TEG-LY30% and conventional D-dimer).

In our study, there were significant differences between the two hypocoagulant and normocoagulant groups for some of TEG^®^6s parameters, although the median values of R, K, the α-angle, and the MA remained within the normal range.

We hypothesized that intraoperative factors such as bleeding or hemodilution could have impacted CCTs but not the TEG^®^6s results, thus emphasizing the potential reliability of TEG^®^6s in certain settings. However, none of the two methods could predict the extent of postoperative bleeding (crucial information for clinicians).

According to previous studies, the relevance of thromboelastography in liver surgeries, particularly in patients with cirrhosis undergoing total hepatectomy or liver transplantation, seems high, especially in optimizing transfusions and managing both bleeding and thrombotic risks [35,36,37,38,39,40,41,42]. Moreover, it could help in the prediction and management of postoperative dysfunction after liver transplantation [43,44,45,46].

Our study has several strengths. This prospective study investigates a group of patients with potentially impaired coagulation. Although thromboelastography has been used in retrospective studies performed in patients with liver disease, to the best of our knowledge, the novel TEG^®^6s has never been investigated in this setting. Furthermore, the direct analysis of the blood samples without preliminary centrifugation and the easy autocleaning process constitute adjunct values, particularly in the ICU. Overall, the testing modality is faster, more straightforward, and ready to use for non-technical operators. However, besides technical and logistic issues in liver-resected patients, who are at risk of hypocoagulant and hypercoagulant situations, TEG^®^6s allows a fast, comprehensive, real-time assessment of the coagulation process. This is paramount when CCTs indicate hypercoagulability or overestimate hypocoagulation. Both conditions are not corroborated by thromboelastography. In patients undergoing liver resection, where coagulation management is critical, TEG^®^6s provides faster, more reliable, and intuitive testing compared to TEG^®^5000. Its automation, bedside availability, and enhanced resistance to external factors make it the optimal choice for guiding hemostatic therapy in real time, ultimately improving patient outcomes and resource efficiency in liver surgery [47,48].

The study has limitations, including being single-center and non-randomized research. The sample size was insufficient, resulting in a potential type II error. As this was the first study evaluating the added value of TEG^®^ 6s data, it is considered a pilot study, so formal sample size calculations were not required. Using Brikett and Day’s guidelines for internal pilot studies [49], a minimum of 20 subjects were needed. To account for dropouts, 34 subjects were included, aiming for 80% power and a 5% significance level concerning the primary endpoint, assuming a moderate effect size. The lack of preoperative TEG tracings did not allow us to determine whether the postoperative discrepancies between TEG and CCTs could be related to some patients’ baseline hypercoagulant profiles. The criteria for hypocoagulability come from the 2007 German guidelines for epidural catheter insertion and removal [50]. Although these guidelines have been used in liver resection [23,51], they may not have been the most suitable for the clinical context explored in this study. Finally, in our study, we detected a longer ICU length of stay in hypocoagulable patients, potentially linked to a greater transfusion load. Even if the implementation of viscoelastic tests was associated with reduced blood product transfusion in other settings, such as liver transplantation patients [37], we are far from demonstrating the beneficial effects on mortality and hospital and the ICU length of stay.

## 5. Conclusions

This study revealed a discrepancy between CCTs and thromboelastographic variables, with CCTs indicating hypocoagulability, while TEG^®^6s suggested normal coagulation following liver resection in oncological patients.

Weak associations were observed between TEG measures of both clot formation and clot strength and conventional fibrinogen level tests, as well as between TEG/CCTs measuring coagulation initiation, TEG, conventional platelet counts, and measurements of fibrinolysis.

This aligns with available evidence suggesting that measures of clotting initiation and speed are challenging to interpret in this cohort, while TEG parameters may be more reliable.

Therefore, based on our results, we suggest the use of perioperative thromboelastography alongside conventional coagulation tests to safely manage transfusions of coagulation products, bearing in mind that only blood clotting assessment allows the detection of true coagulopathy.

## Figures and Tables

**Table 1:** Baseline characteristics and intraoperative parameters. Data are median [interquartile range] or *n* (%). Normal values of thromboelastography variables are wrapped by parentheses that follow parameter name.

Variable	Total Sample (*n* = 46)	NCG Group (*n* = 27)	HCG Group (*n* = 19)	*p* Value
**Baseline characteristics**				
Males	30 (65)	17 (63)	13 (68)	0.762
Age, years	70 [59–75]	69 [59–71]	73 [57–75]	0.467
BMI, kg/m^2^	26 [23–30]	26 [22–30]	27 [25–29]	0.577
ASA 2	33 (72)	20 (74)	13 (68)	0.746
ASA 3	13 (28)	7 (26)	6 (32)	0.746
Cirrhosis	7 (15)	5 (19)	2 (11)	0.682
HCC/cholangiocarcinoma	20 (43)	11 (41)	9 (47)	0.552
Metastatic colorectal cancer	26 (57)	16 (59)	10 (53)	0.766
Anticoagulant therapy *	4 (9)	3 (11)	1 (5)	0.632
Heparin therapy *	0 (0)	0 (0)	0 (0)	-
Antiplatelet therapy *	11 (24)	6 (22)	5 (26)	0.749
Hb *, g/dL	13.3 [11.6–14.6]	13.4 [12.3–14.3]	13.3 [10.8–15.4]	0.817
**Intraoperative parameters**				
Type of surgery				
Minor	27 (59)	17 (63)	10 (53)	0.552
Major	13 (28)	8 (30)	5 (26)	0.806
Minor complex	6 (13)	2 (7)	4 (21)	0.213
Laparotomic	25 (54)	13 (48)	12 (63)	0.377
Laparoscopic	21 (46)	14 (52)	7 (37)	0.377
Surgery duration, min	555 [480–660]	540 [480–600]	600 [540–720]	0.087
PM duration, min	103 [59–143]	95 [53–121]	113 [87–197]	0.078
EBL, mL	500 [300–930]	400 [300–700]	900 [500–1400]	0.002
Fluids, mL	3750 [3000–5000]	3500 [3000–4010]	4500 [3500–6000]	0.019
PRBC-transfused patients	15 (33)	5 (19)	10 (53)	0.025
FFP-transfused patients	2 (4)	0 (0)	2 (11)	0.165
PLT-transfused patients	0 (0)	0 (0)	0 (0)	-

* Preoperative; NCG, normocoagulable; HCG, hypocoagulable; ASA, American Society of Anesthesiology physical status; BMI, body mass index; HCC, hepatocellular carcinoma; Hb, hemoglobin; PM, Pringle maneuver; EBL, estimated blood loss; PRBC, packed red blood cell; FFP, fresh frozen plasma; PLT, platelet.

**Table 2:** Postoperative parameters at ICU admission and 12 h after. Data are median [interquartile range] or *n* (%). Normal values of thromboelastography variables are wrapped by parentheses that follow parameter name.

Variable	Total Sample (*n* = 46)	NCG Group (*n* = 27)	HCG Group (*n* = 19)	*p* Value
**Postoperative parameters at ICU admission**				
Hemoglobin, g/dL	10.8 [9.2–12.0]	11.6 [9.2–12.3]	10.0 [9.2–11.1]	0.284
PLT, ×10^9^/L	159 [107–217]	187 [112–233]	142 [104–172]	0.074
aPTT, s	33.3 [30.1–38.5]	31.0 [27.7–32.8]	39.2 [36.1–43.3]	<0.001
INR	1.31 [1.19–1.39]	1.22 [1.13–1.32]	1.41 [1.32–1.55]	<0.001
PT, s	13.8 [12.7–14.7]	13 [12–14]	15.2 [13.9–16.3]	<0.001
Fibrinogen, mg/dL	247 [214–279]	254 [236–285]	221 [187–258]	0.020
D-dimer	6138 [4372–16,270]	5952 [4238–9880]	8682 [4749–25,055]	0.169
Antithrombin III, %	53 [47–65]	58 [51–72]	49 [40–53]	0.002
Arterial lactate, mmol/L	5.5 [3.0–8.1]	5.1 [2.5–6.6]	6.3 [4.1–11.2]	0.036
CK-R, min (4.6–9.1)	6.2 [5.4–6.6]	5.7 [5.3–6.2]	6.3 [5.8–7.4]	0.021
CK-K, min (0.8–2.1)	1.3 [1.2–1.7]	1.3 [1.2–1.5]	1.4 [1.2–2.7]	0.183
CK α-angle, degrees (63–78)	72.4 [69.9–74.1]	72.5 [71.4–74.2]	71.2 [60.0–74.1]	0.160
CK-MA, mm (52–69)	59.0 [55.3–62.8]	60.8 [57.4–63.5]	56.9 [52.9–61.3]	0.035
CK-LY30, % (0.0–2.6)	0.0 [0.0–0.4]	0.0 [0.0–0.4]	0.0 [0.0–0.5]	0.779
CRT-ACT, s (82–152)	106.6 [97.3–125.3]	97.3 [87.9–116.0]	116.0 [97.3–144.0]	0.003
CRT-R, min (0.3–1.1)	0.6 [0.5–0.8]	0.5 [0.4–0.7]	0.7 [0.5–1.0]	0.003
CRT-K, min (0.8–2.7)	1.8 [1.3–2.0]	1.6 [1.2–1.8]	1.8 [1.4–2.3]	0.158
CRT α-angle, degree (60–78)	71.4 [67.8–74.1]	72.5 [68.6–74.7]	69.5 [63.4–73.0]	0.035
CRT-MA, mm (52–70)	59.4 [55.3–62.1]	60.7 [57.0–62.6]	56.5 [50.4–60.5]	0.026
CRT-LY30, % (0.0–2.2)	0.0 [0.0–0.1]	0.0 [0.0–0.2]	0.0 [0.0–0.1]	0.684
CFF-MA, mm (15–32)	18.8 [16.4–20.8]	19.0 [18.0–21.9]	17.5 [13.6–19.6]	0.038
CFF-A10, mm (15–30)	18.2 [15.5–20.3]	18.7 [16.7–21.1]	15.5 [11.8–18.7]	0.009
**Parameters assessed 12 h after admission to ICU**				
Norepinephrine	10 (22)	3 (11)	7 (37)	0.067
EBL, mL	150 [100–200]	100 [10–200]	170 [150–200]	0.099
PRBC-transfused patients	9 (20)	4 (15)	5 (26)	0.276
FFP-transfused patients	1 (2)	0 (0)	1 (5)	0.413
PLT-transfused patients	0 (0)	0 (0)	0 (0)	-
Fluids, mL	3050 [2100–4300]	2800 [2100–4100]	3200 [2000–4800]	0.409
Arterial lactate, mmol/L	1.5 [1.1–2.1]	1.4 [1.1–2.1]	1.5 [1.4–2.6]	0.198
**Outcome**				
Thromboembolic events °	3 (7)	0 (0)	3 (16)	0.073
ICU LoS, days	1 [1–2]	1 [1–1]	1 [1–2]	0.010
ICU mortality °	0	0	0	**-**

° at 30 days postoperatively; NCG, normocoagulable; HCG, hypocoagulable; EBL, estimated blood loss; PRBC, packed red blood cell; FFP, fresh frozen plasma; PLT, platelet; ICU, intensive care unit; INR, international normalized ratio; aPTT, activated partial thromboplastin time; PT, prothrombin time; CK, citrated kaolin; CRT, citrated rapid thromboelastography; ACT, activated clotting time; CFF, citrated functional fibrinogen; MA, maximum amplitude; LY30, 30 min clot lysis; LoS, length of stay.

**Table 3:** Correlation and linear regression coefficients.

	INR	aPTT	PLT	Fibrinogen	D-Dimer
**Total sample (n = 46)**					
**CK-R**	r = 0.221 (*p* = 0.144)	r = 0.237 (*p* = 0.116)	-	-	
**CK-K**	r = 0.253 (*p* = 0.093)	r = −0.031 (*p* = 0.838)	-	-	
**CK-**α-angle	-	-	r = 0.516 **(*p* < 0.001)**	r = 0.501 **(*p* < 0.001)**	
			r^2^ = 0.065 (*p* = 0.091)	r^2^ = 0.077 (*p* = 0.065)	
**CK-MA**	-	-	r = 0.640 **(*p* < 0.001)**	r = 0.662 **(*p* < 0.001)**	
			r^2^ = 0.419 **(*p* < 0.001)**	r^2^ = 0.484 **(*p* < 0.001)**	
**CRT-R**	**r = 0.647 (*p* < 0.001)**	r = 0.376 **(*p* = 0.011)**	-	-	
	**r^2^ = 0.448 (*p* < 0.001)**	r^2^ = 0.089 (*p* = 0.046)			
**CRT-K**	r = 0.254 (*p* = 0.092)	r = 0.008 (*p* = 0.958)	-	-	
**CRT**-α-angle	-	-	r = 0.413 **(*p* = 0.005)**	r = 0.759 **(*p* < 0.001)**	
			r^2^ = 0.200 (*p* = 0.002)	r^2^ = 0.542 **(*p* < 0.001)**	
**CRT-MA**	-	-	r = 0.618 **(*p* < 0.001)**	r = 0.699 **(*p* < 0.001)**	
			r^2^ = 0.371 **(*p* < 0.001)**	r^2^ = 0.526 **(*p* < 0.001)**	
**CFF-MA**	-	-	-	r = 0.683 **(*p* < 0.001)**	
				r^2^ = 0.514 **(*p* < 0.001)**	
**CFF-A10**	-	-	-	r = 0.751 **(*p* < 0.001)**	
				r^2^ = 0.598 **(*p* < 0.001)**	
**CK-Ly30**					r = −0.232 (*p* = 0.150)
**CRT-Ly30**					r = −0.174 (*p* = 0.284)

INR, international normalized ratio; aPTT, activated partial thromboplastin time; PLT, platelet; CK, citrated kaolin; CRT, citrated rapid thromboelastography; CFF, citrated functional fibrinogen; MA, maximum amplitude; ACT, activated clotting time.

## Data Availability

Data are available on reasonable request from R.G. (e-mail: rita.gaspari@unicatt.it).
